# The Pathology of Fear

**Published:** 1909-05

**Authors:** W. T. Marrs

**Affiliations:** Peoria, Ill.


For Texas Medical Journal.

### The Pathology of Fear.

BY W. T. MARRS, M. D., PEORIA, ILL.

The world is scared. Abject fear in some form dominates the lives of thousands and thousands of individuals. Everybody has fears of some sort. Indulged in normally and with judicious limitations, fear is a good thing and makes us provident and watchful over our future welfare. Without a measure of it we would all be careless, shiftless and purposeless, letting every day and moment pass idly by.

But it is of immoderate and abnormal fear of which I desire to speak briefly. Fear when overindulged in some of its protean forms can make its possessor very unhappy, vacillating in character and in a great measure inhibit his usefulness to others. More than this, fear abnormally developed lowers the vital tone and the natural bodily powers of resistance in such a manner as to invite early disease and decay. If we accept the theory of the neurons and nerve electrification there is nothing calculated to produce greater nerve inhibition or leakage than for an individual to be harassed by nagging and persistent fear. Many persons die simply because they fear they can not get well. It is a well known fact that those who run from disease are the ones more likely to be overtaken by it. Blessed is the doctor who can allay morbid fear, tranquilize a disturbed nervous system and quiet and appease mental unrest.

It is surprising to note the number of our patients who are dominated by tormenting and often groundless fears. Neuroses and psychoses are entirely too numerous nowadays. Neurotic affections seem to be multiplying. What is to blame for it? Fast living explains a good deal. It embraces alcoholic and sexual excesses, the abortion habit, the prevention of conception and many kindred conditions. Dope habits were never so numerous and their habitues are increasing constantly. What these things do not do for the individual they do for his posterity. The immutable law of transmission comes in here and the same nerve impress is passed on down the line.

The psychosis of fear is singled out for a few remarks because it is a thing so often overlooked. People have a reluctance to speak of their morbid fears and fancies even to their doctor. These phobias manifest themselves in divers ways. They usually concern the individual's health and well being and either directly or remotely get around to the fear of death. Sometimes, however, these abnormal fears relate to the little frivolous and foolish things of life. It is very akin to superstitions and folk-lore. Even the educated physician who reads these lines is perhaps a trifle leary about sitting down to a table surrounded by twelve others.

The more common fears are that of lightning, microbes, drowning, falling from a high building, the breaking of elevators, train wrecks, or disease or injury in some one of a thousand and one different forms. Every woman has a sort of unexpressed fear that she will acquire the disease which some lady friend is suffering from. And isn't it a bit strange how often she gets it? Thousands live in mortal fear of appendicitis and so very often acquire it in the most modern and approved form. Of course I am a nasty heretic for saying it, but appendicitis in the great majority of cases seems to me to be only an overworked imagination, aided and abetted by the medical man. The enormous drug and nostrum business is based upon people's fear of disease. If we could eliminate morbid fear and in some measure forget that we possess vital organs that are likely to go wrong we would be better off. The cases of hydrophobia which I have ever seen were only hysteria intensified by the obsession that the individuals were suffering from this horrible malady. Hydrophobia perhaps exists, but I have never seen a case.

This subject is sufficiently expansive for the writing of a volume on it. I could grow reminiscent and embellish the idea with a number of illustrations from my own experience. In this short paper I desire only to emphasize the fact that we can do a world of good if we take the pains to diagnose morbid and uncanny fears and go about the task of obliterating them in the right manner.

---

March 24th, 1909.

*Dr. F. E. Daniel, Austin, Texas.*

DEAR DOCTOR: I enclose herewith money order for two dollars. Your journal, as a fearless and outspoken advocate of what you believe to be right, fills an important place in medical journalism. You are a hard hitter and, no doubt, dearly love a "scrap." Well, there are plenty of abuses which creep into our "noble profession" that need your attention, and some of them seem to be claiming a part of your attention at this particular time. Fraternally yours,

J. F. MCCARTY, M. D.,
Comanche, Texas.

